# Fuzzy-Adaptive ESO Control for Dual Active Bridge Converters

**DOI:** 10.3390/s26010048

**Published:** 2025-12-20

**Authors:** Ju-Hyeong Seo, Sung-Jin Choi

**Affiliations:** Department of Electrical, Electronic and Computer Engineering, University of Ulsan, Ulsan 44610, Republic of Korea; chris3342@mail.ulsan.ac.kr

**Keywords:** active disturbance rejection control (ADRC), adaptive bandwidth, DC microgrids (DCMG), dual active bridge (DAB), extended state observer (ESO), fuzzy logic

## Abstract

In converter-dominated direct-current microgrids, severe load transients can cause large voltage deviations on the common direct-current bus. To mitigate this, an energy storage system is typically employed, and an isolated bidirectional dual active bridge converter is commonly used as the power interface. Therefore, the controller must ensure robust transient performance under step-load conditions. This paper proposes an active disturbance rejection control framework that adaptively adjusts the bandwidth of an extended state observer using fuzzy logic. The proposed observer increases its bandwidth during transients—based on the estimation error—to accelerate disturbance compensation, while decreasing the bandwidth near steady state to suppress noise amplification. This adaptive tuning alleviates the fixed-bandwidth trade-off between transient speed and noise sensitivity in ESO-based regulation. Hardware experiments under load-step conditions validate the method: for a load increase, the peak voltage undershoot and settling time are reduced by 22% and 48.9% relative to a proportional–integral controller, and by 20% and 36.1% relative to a fixed-bandwidth observer. For a load decrease, the peak overshoot and settling time are reduced by 27.9% and 49.5% compared with the proportional–integral controller, and by 20.5% and 25% compared with the fixed-bandwidth observer.

## 1. Introduction

Recently, driven by carbon-neutrality targets and the efficiency requirements of power-electronics-dominated systems, DC microgrids (DCMGs) are gaining traction across applications such as data centers, transportation electrification, and industrial facilities [1]. Compared with ac distribution, DC architectures avoid frequency and phase synchronization requirements, reduce issues related to reactive-power flows and harmonics/unbalances, and interface naturally with renewable sources, power-electronic loads, and energy storage systems (ESS) [1,2]. In particular, in islanded or weakly grid-tied DCMGs, rapid variations in load and generation can induce deviations of the common DC-bus voltage; thus, tight regulation and fast restoration to the nominal reference become key control objectives for power quality and reliability [3,4,5]. To mitigate these power imbalances, an ESS is commonly integrated to buffer fast dynamics [6]. As a bidirectional power interface between the ESS and the DC bus, the dual active bridge (DAB), an isolated bidirectional DC/DC converter, is widely used [7,8]. The DAB offers high-frequency galvanic isolation, high power conversion efficiency over a wide operating range, and flexible power flow control through various modulation techniques such as single-phase-shift (SPS) and triple-phase-shift (TPS) [9,10,11,12,13,14]. Moreover, accurate average and discrete models have been established [11], along with frequency-domain zero-voltage switching (ZVS) boundary analysis [12] and phase-shift control methods for achieving fast dynamic response [13,14]. These modulation and control design guidelines have been systematically presented, and their reliability in high-power applications has been experimentally validated [11,12,13,14]. However, in real DCMG environments, superimposed uncertainties—including bus-impedance variations, model mismatch, sensor noise and delay, and abrupt step-like load changes—make an inherent trade-off between the robustness and dynamic performance of the DAB controller unavoidable [3,10].

Traditionally, single-voltage-loop PI controllers are designed for a specific operating point. Therefore, under rapid load transients, the operating point shifts away from the design point, making it difficult to maintain optimal performance [14,15]. Model predictive control (MPC) offers the advantage of fast transient response; however, it has inherent limitations, including a high computational burden in high-switching-frequency and high-speed sampling environments, as well as sensitivity to model mismatches [16,17,18,19,20]. Conventional sliding mode control (SMC) offers high robustness and fast responsiveness; however, due to its discontinuous switching control, chattering can occur, which may degrade system performance and cause potential damage to mechanical components [21,22]. Therefore, active disturbance rejection control (ADRC), which reduces dependence on detailed models while maintaining fast transient response and robustness, has attracted attention as an alternative [23]. The core of ADRC, the extended state observer (ESO), can compensate for model uncertainties and disturbances by treating them as a single total disturbance, thereby simplifying the control structure [24,25]. However, ESO inherently presents a trade-off between dynamic performance and noise based on the observer bandwidth; increasing the bandwidth improves dynamic performance but simultaneously amplifies noise [8,26,27,28].

To address these limitations, this paper proposes a fuzzy logic-based ESO bandwidth adjustment method for the DAB converter. In this approach, fuzzy logic operates based on the estimation error to adaptively adjust the observer bandwidth of the ESO in real time. This method mitigates the drawbacks of the strong dependence of MPC on models and parameters, the fixed-gain trade-off of ADRC, and chattering in SMC, while enhancing both power quality and reliability under severe step-load transients in converter-dominated DC microgrids.

The main contributions of this paper are summarized as follows:

- A unified frequency-domain analysis of the fixed-bandwidth ESO for DAB voltage regulation is presented, clarifying the trade-off between low-frequency disturbance rejection (under operating-point/model mismatch) and high-frequency noise amplification.
- A fuzzy logic-based adaptive ESO (FESO) is proposed to tune the observer bandwidth online using the ESO estimation error, increasing it only during transients for faster disturbance compensation while reducing it near steady state to mitigate noise sensitivity.
- The proposed method is validated via sensitivity-based robustness analysis under operating-point variations and hardware step-load experiments, demonstrating reduced voltage deviation and shorter settling time compared with a PI controller and a conventional fixed-bandwidth ESO.

The structure of this paper is as follows: Section 2 discusses the principles of ADRC. Section 3 presents the DAB converter controller design methodology and explains the ESO bandwidth trade-off. Section 4 describes the proposed fuzzy logic-based bandwidth adjustment method. Section 5 verifies the performance of the proposed method through hardware experiments. Section 6 discusses the scope of the proposed local controller and future research directions that consider communication constraints in distributed extensions. Finally, Section 7 concludes this paper.

## 2. Review of the ADRC Framework

Active disturbance rejection control (ADRC) models an *n*-th order system as an ideal *n*-th order integrator between the input and output, and separates it into a total disturbance that includes internal parameter uncertainties and external disturbances as shown in (1):

(1) $$y^{\left(n\right)}\left(t\right)=f\left(t\right)+bu\left(t\right).$$

(1) can be expressed in a state-space form with an extended structure that includes the disturbance term. To estimate the total disturbance f(t), the extended model can be reconstructed in the form of a Luenberger observer as shown in (2):

(2) $$\begin{array}{cc} \dot{z} & =Az+Bu+L(\hat{y}-Cz)\\ \tilde{y} & =Cz \end{array}.$$

Here, $\hat{y}$ denotes the actual output, and $\tilde{y}$ represents the estimated output. The observer gain reacts to the difference between the measured output and the estimated value. Using this structure, an ESO (extended state observer) is constructed to estimate the lumped disturbance term.

The input u(t) for compensating the estimated disturbance can be expressed as

(3) $$u\left(t\right)=\frac{u_{0}\left(t\right)-z_{n+1}\left(t\right)}{b}.$$

If the disturbance is perfectly estimated such that $z_{n+1}\left(t\right)=f\left(t\right)$, substituting (3) into (1) yields the following simple *n*-th order integrator form:

(4) $$y^{\left(n\right)}\left(t\right)=u_{0}\left(t\right).$$

The observer gains $\beta_{i}$ are commonly designed via pole placement using a single observer bandwidth parameter $\omega_{o}$ as follows:

(5) βi=n+1iωoi.

As shown in (6), all observer poles are determined by the observer bandwidth $\omega_{o}$:

(6) $$s^{n+1}+\beta_{1}s^{n}+\beta_{2}s^{n-1}+\cdots +\beta_{n+1}={\left(s+\omega_{o}\right)}^{n+1}.$$

As a result, the roots of the characteristic equation are located at $-\omega_{o}$, which defines the dynamic performance of the observer:

(7) $${\left(s+\omega_{o}\right)}^{n+1}=0.$$

As the poles move farther left in the left-half plane (LHP), the observer bandwidth increases, leading to faster convergence of the estimation error to zero. However, if $\omega_{o}$ becomes excessively large, the system becomes sensitive to noise and may suffer from limitations due to the sampling frequency. The detailed derivation of the ESO structure and pole-placement gain design is provided in Appendix A.

## 3. ADRC for the DAB Converter and Its Limitations

### 3.1. Controller Design for the DAB Converter

**Figure 1:**
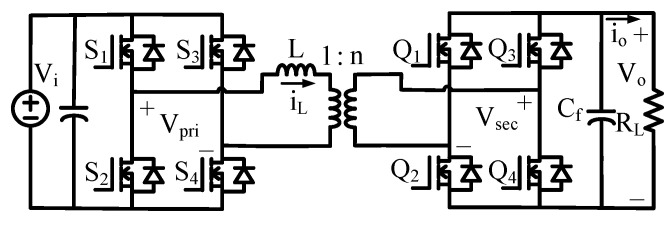
Schematic of the DAB converter.

The DAB (Dual Active Bridge) converter consists of full-bridge circuits on both the input and output sides as shown in Figure 1, and these are connected through a transformer. According to [10], the reduced-order model is employed in this paper since it lowers the model complexity while still ensuring high large-signal and small-signal accuracy. In the reduced-order model, the system is approximated as a first-order transfer function with a single dominant pole. The resulting transfer function can be expressed as follows:

(8) $$G_{vd}\left(s\right)=\frac{{\hat{v}}_{o}}{\hat{d}}=\frac{NV_{i}\left(1-2D\right)}{2f_{s}L}\cdot \frac{R_{L}}{R_{L}C_{f}s+1}.$$

Here, *N* is the turns ratio of the transformer, $V_{i}$ is the input voltage, and *D* is the phase shift ratio, which typically ranges from 0 to 0.5. $R_{L}$ denotes the load resistance, $f_{s}$ is the switching frequency, $C_{f}$ is the output capacitance, and *L* is the series inductance.

To design the ESO, the transfer function in (8) is transformed into the time domain as

(9) $$\frac{d{\hat{v}}_{o}}{dt}=-\frac{{\hat{v}}_{o}}{R_{L}C_{f}}+\frac{NV_{i}\left(1-2D\right)}{2f_{s}LC_{f}}\cdot \hat{d}\left(t\right).$$

Considering both internal and external uncertainties as a total disturbance, the system can be extended as

(10) $$\frac{d{\hat{v}}_{o}}{dt}=-\frac{{\hat{v}}_{o}}{R_{L}C_{f}}+\left(b-b_{0}\right)\hat{d}\left(t\right)+b_{0}\hat{d}\left(t\right)$$

If we define the total disturbance as

(11) $$f\left(t\right)=-\frac{{\hat{v}}_{o}}{R_{L}C_{f}}+\left(b-b_{0}\right)\hat{d}\left(t\right)$$

Then the structure becomes identical to that of (1).

Here, *b* represents the actual DAB converter parameter, and $b_{0}$ is the nominal parameter used in the design, which can be expressed as

(12) $$b_{0}=\frac{N_{0}V_{i0}\left(1-2D_{0}\right)}{2f_{s0}L_{0}C_{f0}}.$$

The input d(t) is defined by (3). If the estimated disturbance is denoted as $\tilde{f}\left(t\right)$, then $\hat{d}\left(t\right)$ can be expressed accordingly as

(13) $$\hat{d}\left(t\right)=\frac{{\hat{d}}_{0}\left(t\right)-\tilde{f}\left(t\right)}{b_{0}}.$$

Therefore, the ESO for the DAB converter can be derived from (A6) and is formulated as follows:

(14) $$\begin{array}{cc} {\dot{\tilde{v}}}_{o} & =\tilde{f}+\beta_{1}\left({\hat{v}}_{o}-{\tilde{v}}_{o}\right)+b_{0}\hat{d}\\ \dot{\tilde{f}} & =\beta_{2}\left({\hat{v}}_{o}-{\tilde{v}}_{o}\right). \end{array}$$

**Figure 2:**
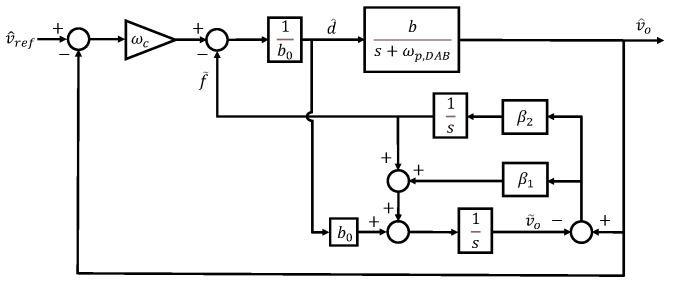
Block diagram of ESO system.

Here, ${\tilde{v}}_{o}$ represents the estimated output voltage. If the disturbance is effectively estimated through the observer, the system can be simplified to a first-order integrator form as described in (4). Accordingly, control can be achieved using a simple proportional controller with gain $\omega_{c}$. The structure of this observer can be illustrated using a block diagram as shown in Figure 2.

### 3.2. Limitation of the Conventional Method

By transforming (14) into the *s*-domain, the estimated disturbance value $\tilde{F}\left(s\right)$ can be expressed as

(15) $$\tilde{F}\left(s\right)=\frac{\beta_{2}s}{s^{2}+\beta_{1}s+\beta_{2}}{\hat{V}}_{o}\left(s\right)-\frac{\beta_{2}b_{0}}{s^{2}+\beta_{1}s+\beta_{2}}\hat{D}\left(s\right).$$

**Figure 3:**
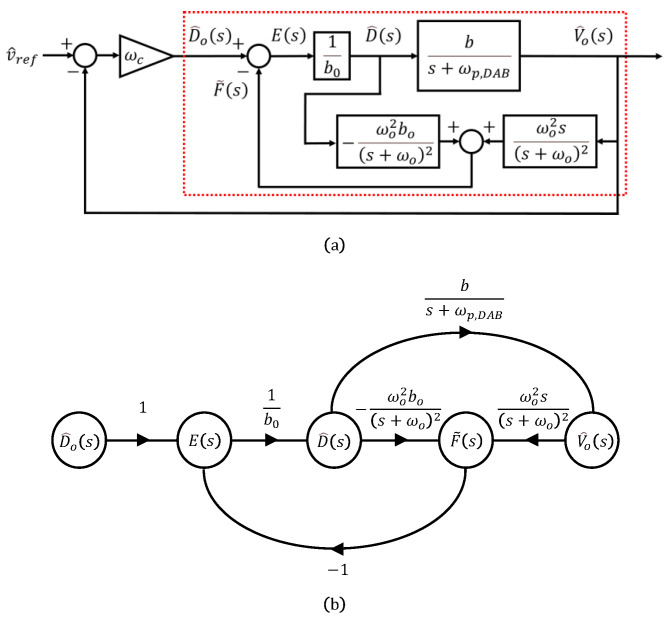
ADRC for DAB: (**a**) simplified diagram, (**b**) signal-flow graph for limitation analysis. The red dotted box indicates the ESO-based disturbance estimation and compensation subsystem.

If we choose $\beta_{1}=2\omega_{o}$ and $\beta_{2}=\omega_{o}^{2}$, the observer gains β can be defined in terms of the observer bandwidth $\omega_{o}$, as shown in (5). This relationship can also be represented using a block diagram, as illustrated in Figure 3a.

By converting the red boxed region in the block diagram into a signal-flow graph as shown in Figure 3b and applying Mason’s gain rule, the output ${\hat{V}}_{o}\left(s\right)$ with respect to the input ${\hat{D}}_{o}\left(s\right)$ can be expressed as follows:

(16) $$G_{vdo}\left(s\right)=\frac{{\hat{V}}_{o}\left(s\right)}{{\hat{D}}_{o}\left(s\right)}=\frac{1}{K+\frac{2}{A}}\cdot \frac{1}{s}\cdot \frac{N_{1}\left(s\right)}{D_{1}\left(s\right)}$$

where,

(17) $$N_{1}\left(s\right)=1+\frac{1}{Q_{o}}\;\left(\frac{s}{\omega_{o}}\right)+{\left(\frac{s}{\omega_{o}}\right)}^{2}$$

(18) $$D_{1}\left(s\right)=1+\frac{1}{Q_{n}}\;\left(\frac{s}{\omega_{n}}\right)+{\left(\frac{s}{\omega_{n}}\right)}^{2}.$$

The transfer function consists of an overall gain, an integrator, and second-order systems in both the numerator and the denominator. Here, we introduce two design parameters: $K=\frac{b}{b_{0}}$ represents the ratio of the designed parameter to the actual parameter, indicating how much the designed value differs from the actual value, and $A=\frac{\omega_{o}}{\omega_{p,\mathrm{DAB}}}$ denotes the ratio of the observer bandwidth to the pole frequency of the DAB, indicating how many times greater the observer’s bandwidth is than the converter’s pole frequency. The natural frequency of the denominator is given by

(19) $$\omega_{n}=\omega_{o}\sqrt{K+\frac{2}{A}},$$

while the quality factors are usually defined as $Q_{o}=0.5$ for the numerator and (20) for the denominator.

(20) $$Q_{n}=\frac{\sqrt{K+\frac{2}{A}}}{2\left(1+\frac{1}{2A}\right)}.$$

**Figure 4:**
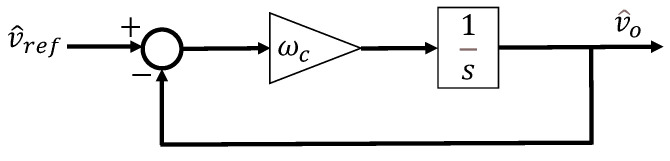
ADRC for DAB in the ideal case.

In the ideal case, the nominal and actual parameters of the DAB converter are perfectly matched, resulting in K=1, and the observer bandwidth becomes infinitely large, leading to A→∞. Consequently, the natural frequency of the denominator $\omega_{n}$ becomes equal to the natural frequency of the numerator $\omega_{o}$, and the quality factor of the denominator also becomes equal to that of the numerator. As a result, the second-order terms result in cancellation, and the overall gain term $\frac{1}{\sqrt{K+\frac{2}{A}}}$ converges to 1. Therefore, (16) takes the form of a simple integrator, and with the proportional gain $\omega_{c}$, it can be represented as shown in Figure 4, indicating that the larger the observer bandwidth, the better the disturbance estimation performance.

Conversely, when the observer bandwidth approaches zero, the quality factor associated with the denominator (20) becomes less than 0.5. Since $Q_{n}<0.5$ in this case, the characteristic denominator (18) has two distinct real roots, which are given by

(21) $$s_{1,2}=-\frac{\omega_{n}}{2Q_{n}}\pm \frac{\omega_{n}}{2Q_{n}}\sqrt{1-4Q_{n}^{2}}.$$

**Figure 5:**
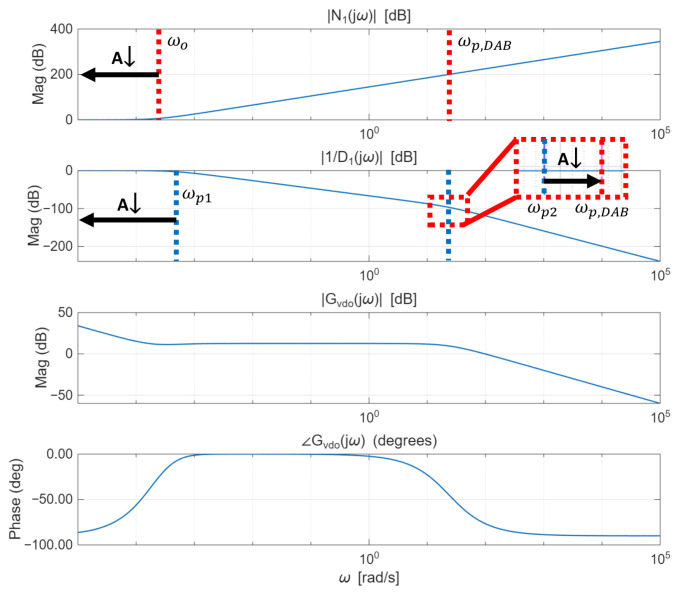
Bode plot of ${\hat{V}}_{o}$ with respect to ${\hat{D}}_{o}$ under a small observer bandwidth, where $A=\omega_{o}/\omega_{p,\mathrm{DAB}}$ denotes the observer-bandwidth ratio. The red dotted line indicates the pole frequency of $|N_{1}\left(j\omega \right)|$, and the blue dotted line indicates the pole frequency of $\left|1/D_{1}\left(j\omega \right)\right|$.

As *A* decreases, substituting (19) and (20) into (21) and simplifying shows that $s_{1}$ approaches 0 and $s_{2}$ approaches $-\omega_{p,\mathrm{DAB}}$. Therefore, the first pole frequency $\omega_{p1}$ decreases toward zero, and the second pole $\omega_{p2}$ settles at $\omega_{p,\mathrm{DAB}}$. This behavior can be analyzed through the Bode plot shown in Figure 5.

In Figure 5, $\left|N_{1}\left(j\omega \right)\right|$ represents the magnitude of the numerator term of the second-order system in (16), whereas $\left|1/D_{1}\left(j\omega \right)\right|$ denotes the magnitude of the second-order pole factor (i.e., the inverse of the normalized denominator). In addition, $\left|G_{vdo}\left(j\omega \right)\right|$ and $∠G_{vdo}\left(j\omega \right)$ denote the overall magnitude and phase of (16), respectively.

The second-order numerator increases at a rate of 40 dB/dec around $\omega_{o}$, while the first pole $\omega_{p1}$ in the denominator causes a decrease of 20 dB/dec. Subsequently, the second pole $\omega_{p2}$, located near the DAB converter’s pole frequency $\omega_{p,\mathrm{DAB}}$, contributes an additional 40 dB/dec attenuation. When an integrator and an overall gain are added as seen in the magnitude of $G_{vdo}\left(j\omega \right)$, a flat region appears in the low-frequency range up to the second pole of the denominator. During this region, the overall gain converges to zero. Afterward, the response begins to decrease at a rate of 20 dB/dec. This indicates the formation of a mid-band gain at the crossover frequency, during which the phase margin becomes almost 180 degrees. Consequently, the low-frequency loop gain decreases, degrading disturbance rejection at low frequencies.

From Figure 3b, using Mason’s rule, the relationship between the observer bandwidth and noise can be described by the output-to-disturbance transfer function (22):

(22) $$G_{fv}\left(s\right)=\frac{\tilde{F}\left(s\right)}{{\hat{V}}_{o}\left(s\right)}=\frac{\omega_{p,\mathrm{DAB}}}{K+\frac{2}{A}}\cdot \frac{N_{2}\left(s\right)}{D_{2}\left(s\right)}$$

where

(23) $$N_{2}\left(s\right)=1+\frac{s}{\omega_{p,\mathrm{DAB}}}$$

(24) $$D_{2}\left(s\right)=1+\frac{1}{Q_{n}}\;\left(\frac{s}{\omega_{n}}\right)+{\left(\frac{s}{\omega_{n}}\right)}^{2}.$$

**Figure 6:**
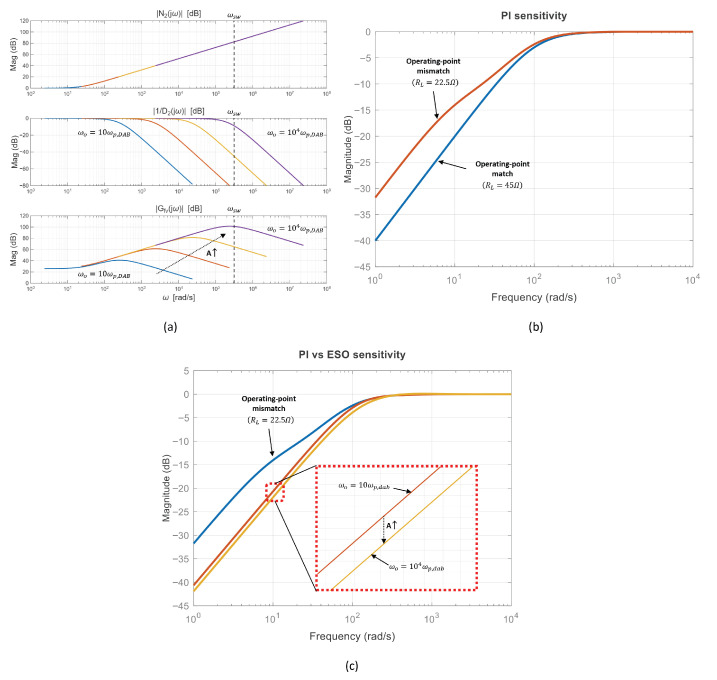
Unified theoretical comparison illustrating the fixed-bandwidth ESO trade-off and the expected benefit of the proposed FESO. (**a**) Bode plot of the disturbance–estimation dynamics versus $A=\omega_{o}/\omega_{p,\mathrm{DAB}}$ (K=1). Solid curves compare A=10, 100, 1000, and 10,000; the dotted arrow indicates increasing *A*, and the vertical dashed line denotes $\omega_{sw}$. (**b**) Sensitivity–magnitude comparison for a PI controller under operating-point variation: matched case ($R_{L}=45\;\Omega$; design point) vs. mismatched case ($R_{L}=22.5\;\Omega$). (**c**) Controller comparison under the same mismatched condition ($R_{L}=22.5\;\Omega$): PI vs. fixed-bandwidth ESO with different observer bandwidths ($\omega_{o}=10\omega_{p,\mathrm{dab}}$, $10^{4}\omega_{p,\mathrm{dab}}$).

The output voltage ${\hat{V}}_{o}\left(s\right)$ consists of various frequency components, including ripple, load variations, and noise. The observer estimates the disturbance $\tilde{F}\left(s\right)$ based on this signal. Therefore, (22) allows the analysis of the observer’s disturbance estimation characteristics in the frequency domain. Assuming the perfectly matched case (K=1), the resulting Bode plot is shown in Figure 6a. The Bode plot illustrates the cases where *A* is 10, 100, 1000, and 10,000, and $\omega_{sw}$ denotes the switching frequency. Here, $\left|N_{2}\left(j\omega \right)\right|$ denotes the magnitude of the numerator term, and $\left|1/D_{2}\left(j\omega \right)\right|$ represents the magnitude contribution of the second-order denominator term. In addition, $\left|G_{fv}\left(j\omega \right)\right|$ indicates the overall magnitude of (22).

The magnitude of $N_{2}\left(j\omega \right)$ increases at 20dB/dec for frequencies above $\omega_{p,\mathrm{DAB}}$. The contribution of the second-order denominator, $\left|1/D_{2}\left(j\omega \right)\right|$, corresponds to two real poles; after the first pole the slope decreases at 20dB/dec, and after the second pole it decreases at 40dB/dec. As *A* increases, the quality factor $Q_{n}$ approaches 0.5, and the two real poles migrate toward $A\;\omega_{p,\mathrm{DAB}}$ according to (17) and (19), becoming nearly a repeated root. Consequently, the ESO disturbance–estimation magnitude $|G_{fv}\left(j\omega \right)|$ increases at 20dB/dec beyond $\omega_{p,\mathrm{DAB}}$, exhibits a peak near $A\;\omega_{p,\mathrm{DAB}}$, and then decreases at 20dB/dec. As shown in Figure 6a, when $\omega_{o}=10^{4}\;\omega_{p,\mathrm{DAB}}$, the natural frequency $\omega_{n}$ lies near the switching frequency, causing the observer to treat high-frequency noise as a disturbance. As a result, the system becomes highly susceptible to noise.

Therefore, if the bandwidth is too low, disturbances cannot be accurately estimated. On the other hand, if the bandwidth is too high, the observer becomes more vulnerable to noise. This indicates a trade-off relationship between estimation accuracy and noise robustness. Therefore, ref. [29] recommends setting the observer bandwidth $\omega_{o}$ to be 3 to 10 times the control bandwidth $\omega_{c}$. The closed-loop transfer function in Figure 4 can be expressed as

(25) $$\frac{1}{\;1+\frac{s}{\omega_{c}}\;}.$$

Considering the 2% settling time criterion, the gain $\omega_{c}$ can be determined using

(26) $$\omega_{c}\approx \frac{4}{T_{\mathrm{settle}}}.$$

### 3.3. Sensitivity-Based Robustness Analysis Under Operating-Point Variations

As shown in the reduced-order model of (8), the DAB voltage plant exhibits a dominant pole at $\omega_{p}=1/\left(R_{L}C_{f}\right)$, which varies significantly with the load resistance. As the load becomes lighter ($R_{L}$ increases), $\omega_{p}$ decreases and the plant approaches an integrator. In particular, for a resistive load, the no-load operation is reported to depict a conservative (worst-case) condition for voltage-controller design [30]. Therefore, a fixed-gain controller designed at a nominal operating point may experience performance degradation when the plant deviates from the design point.

To quantify such robustness characteristics in a unified frequency-domain manner, we employ the sensitivity function

(27) $$S\left(s\right)≜\frac{1}{1+L(s)},\;L\left(s\right)=C\left(s\right)P\left(s\right)$$

where P(s) and C(s) denote the plant and controller transfer functions, respectively. Since load variations and reference changes are primarily low-frequency phenomena, the low-frequency magnitude of |S(jω)| directly indicates disturbance-rejection capability: a smaller |S(jω)| at low frequencies corresponds to the improved attenuation of load-induced output-voltage deviations.

Figure 6b compares the sensitivity of a fixed PI controller designed at a light-load operating point ($R_{L}=45\;\Omega$) when the plant is evaluated at the design point ($R_{L}=45\;\Omega$) and at a heavier-load condition ($R_{L}=22.5\;\Omega$). Although the PI controller achieves the intended bandwidth at the nominal condition, the operating-point shift changes the dominant pole location ($\omega_{p}=1/\left(R_{L}C_{f}\right)$) and the effective loop gain, which can increase the low-frequency sensitivity magnitude. This implies degraded rejection of load disturbances when the plant departs from the nominal design point.

Figure 6c compares the PI controller and the ESO-based controllers under the same mismatch condition (controllers designed at $R_{L}=45\;\Omega$ while the plant is evaluated at $R_{L}=22.5\;\Omega$). Two bandwidth settings, $\omega_{o}=10\omega_{p,\mathrm{dab}}$ and $\omega_{o}=10^{4}\omega_{p,\mathrm{dab}}$, are considered to illustrate the effect of $\omega_{o}$ on low-frequency disturbance rejection. As $\omega_{o}$ increases, the low-frequency sensitivity magnitude can be reduced, indicating improved attenuation of load-induced output-voltage deviations. On the other hand, the noise-related drawback of an excessively large $\omega_{o}$ is more directly captured by the disturbance–estimation characteristics in Figure 6a, where $\omega_{n}$ approaching $\omega_{sw}$ causes high-frequency ripple and noise to be treated as disturbances. These observations motivate the proposed fuzzy-bandwidth ESO, which increases $\omega_{o}$ only during transient load steps and decreases $\omega_{o}$ near steady state to avoid unnecessary noise sensitivity.

## 4. Proposed Fuzzy-Bandwidth ESO

As discussed in Section 3, a limitation of the conventional ESO is that its bandwidth is fixed. If the bandwidth is designed to be large in pursuit of fast response, the observer becomes more sensitive to noise. Conversely, if the bandwidth is reduced to enhance noise immunity, the disturbance estimation performance deteriorates. This reveals an inherent trade-off between the accuracy of the estimate and the robustness to noise. To overcome this limitation, this paper proposes a fuzzy logic-based ESO that adjusts the observer bandwidth in real time according to the magnitude of the estimation error.

### Membership Function

The membership function in fuzzy logic is designed based on the voltage error between the actual output voltage and the voltage estimated by the ESO, and it satisfies the following rules:

- Rule 1: If $e_{r}$ is VL (Very Low), then $n_{\mathrm{fuzzy}}$ is $\mu_{VL}$
- Rule 2: If $e_{r}$ is L (Low), then $n_{\mathrm{fuzzy}}$ is $\mu_{L}$
- Rule 3: If $e_{r}$ is M (Medium), then $n_{\mathrm{fuzzy}}$ is $\mu_{M}$
- Rule 4: If $e_{r}$ is H (High), then $n_{\mathrm{fuzzy}}$ is $\mu_{H}$
- Rule 5: If $e_{r}$ is VH (Very High), then $n_{\mathrm{fuzzy}}$ is $\mu_{VH}$

Here, $e_{r}$ represents the relative error between the estimated voltage ${\tilde{v}}_{o}$ from the observer and the actual output voltage ${\hat{v}}_{o}$, which is quantitatively defined as

(28) $$e_{r}=\frac{|{\hat{v}}_{o}-{\tilde{v}}_{o}|}{V_{\mathrm{ref}}}\times 100\%.$$

In (28), the numerator $|{\hat{v}}_{o}-{\tilde{v}}_{o}|$ represents the absolute error between the actual output voltage ${\hat{v}}_{o}$ and the estimated voltage ${\tilde{v}}_{o}$ from the observer. The denominator $V_{\mathrm{ref}}$ corresponds to the reference output voltage that the system aims to achieve. This allows the absolute error to be expressed as a percentage of the system reference target, allowing the design criteria to be set more intuitively and clearly.

**Figure 7:**
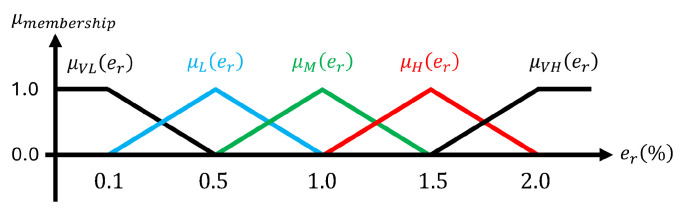
Membership function.

In this paper, the error range is divided into five regions for bandwidth design, as shown in Figure 7. The error magnitude is classified into VL (Very Low), L (Low), M (Medium), H (High), and VH (Very High), depending on the output response of the system and the allowable noise range. First, when the error is within 0.1%, it is regarded as noise, where the signal mainly reflects sensor noise rather than actual disturbance, and the bandwidth is minimized to suppress noise amplification. If the error is between 0.1% and 0.5%, it is defined as the steady-state error region, representing small residual errors in which noise suppression remains a priority. An error between 0.5% and 1% is classified as the internal disturbance region, typically caused by parameter variations, and the bandwidth is moderately increased to compensate for model mismatch. An error between 1% and 2% is regarded as the external disturbance region, associated with load variations or bus impedance changes, requiring faster response through larger bandwidth. Finally, when the error exceeds 2%, it is defined as the transient region, and the bandwidth is maximized to achieve rapid disturbance rejection.

To ensure minimal computational load and latency in real-time applications, the weighted average defuzzification method is used to determine $n_{\mathrm{fuzzy}}$ as defined in (29):

(29) $$\begin{array}{cc} n_{\mathrm{fuzzy}}\; & =\frac{\mu_{\mathrm{VL}}n_{\mathrm{VL}}+\mu_{L}n_{L}+\mu_{M}n_{M}+\mu_{H}n_{H}+\mu_{\mathrm{VH}}n_{\mathrm{VH}}}{\mu_{\mathrm{VL}}+\mu_{L}+\mu_{M}+\mu_{H}+\mu_{\mathrm{VH}}} \end{array}.$$

The value of $n_{\mathrm{fuzzy}}$ calculated from (29) is confined to the range $\left[n_{\mathrm{VL}},\;n_{\mathrm{VH}}\right]$ and is employed as a gain to determine the observer bandwidth $\omega_{o}$, which is defined by

(30) $$\omega_{o}=n_{\mathrm{fuzzy}}\;\omega_{c}.$$

**Figure 8:**
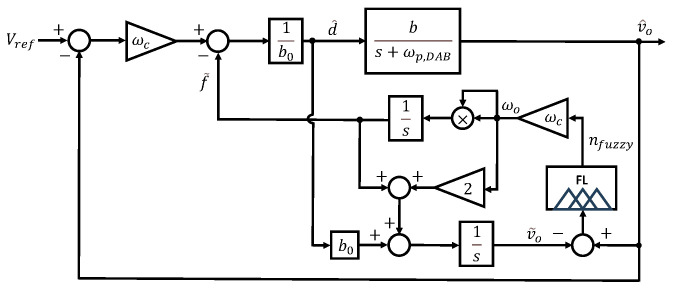
Block diagram of the proposed FESO.

The proposed FESO block diagram is illustrated in Figure 8. As shown in the figures, FESO can dynamically adjust the observer bandwidth in real time. This allows the bandwidth to increase as needed without being significantly affected by noise. Using the error between the actual output and the estimated output of the observer as input to the fuzzy logic, the observer bandwidth can be adaptively adjusted. This dynamic adjustment mechanism enables the proposed FESO to effectively overcome the limitations of conventional fixed-bandwidth ESOs.

## 5. Hardware Results

**Figure 9:**
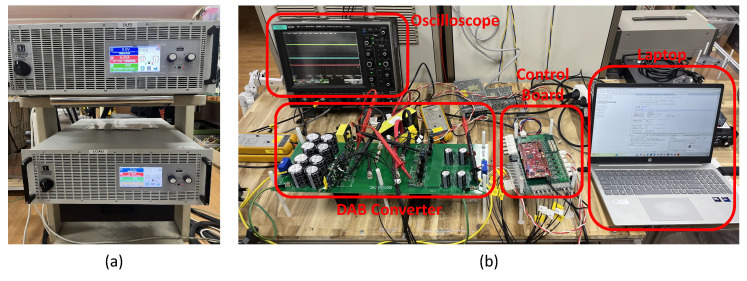
Hardware configuration: (**a**) bidirectional power supplies, (**b**) overall view of the DAB converter hardware setup.

The hardware configuration is shown in Figure 9b. The main parameters of the DAB converter are listed in Table 1, and the control algorithm is implemented on a DSP control platform (TMS320F28379D, Texas Instruments, Dallas, TX, United States). As shown in Figure 9a, two bidirectional power supplies are used at the input and output sides: one is configured as a source, and the other as an electric load to implement the step-load condition.

The hardware test was conducted under an output-voltage-controlled condition of 130 V with two step-load change scenarios. In the first scenario, the load was changed from 45Ω to 22.5Ω, causing the output power to increase from 375 W to 750 W. In the second scenario, the load was changed from 22.5Ω to 45Ω, causing the output power to decrease from 750 W to 375 W. For performance comparison with the proposed FESO, a conventional ESO and a PI controller were implemented. In the case of the conventional ESO, to achieve a settling time of 40 ms, the control bandwidth $\omega_{c}$ was set to 100rad/s using (26), and the ESO bandwidth $\omega_{o}$ was set to 300rad/s, three times the control bandwidth, which is typically accepted. The PI controller was designed using (8) with the pole–zero cancellation method to achieve the same settling time and a phase margin of $90^{{}^{\circ}}$.

**Figure 10:**
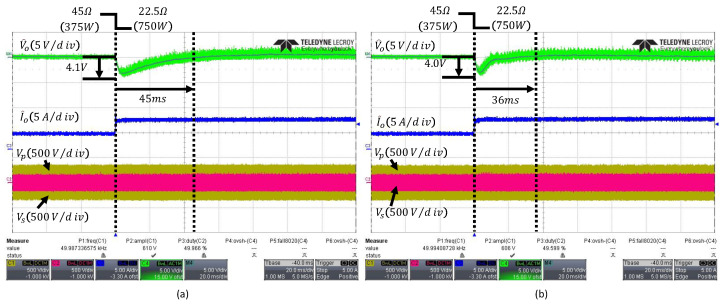
Transient responses to a load step from 45Ω to 22.5Ω: (**a**) PI controller, (**b**) conventional fixed-bandwidth ESO with $\omega_{o}=300\;\mathrm{rad}/s$. The arrows indicate the peak undershoot and the settling time.

Figure 10 shows the waveforms of the output voltage, output current, and the primary- and secondary-side voltages of the PI controller and the conventional ESO when the load changes from 45Ω to 22.5Ω. Figure 10a presents the waveforms of the PI controller under a load change, showing a voltage undershoot of 4.1V and a settling time of 45ms. Figure 10b presents the waveforms of the conventional ESO, which exhibits a comparable voltage undershoot of 4.0V but achieves a shorter settling time of 36ms, approximately 9ms faster than the PI controller. In addition, since the PI controller uses fixed gains, it cannot account for discrepancies between the parameters used in the design and the actual model, leading to degraded transient performance compared with the conventional ESO.

**Figure 11:**
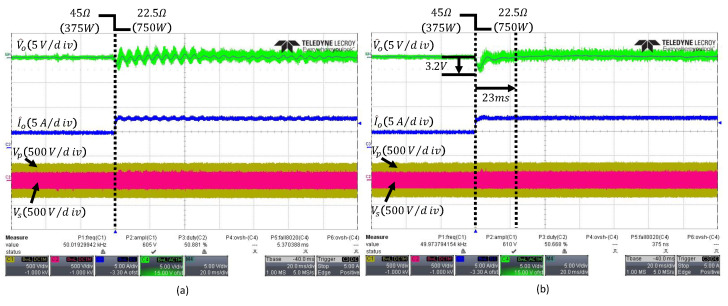
Transient responses under load change from 45Ω to 22.5Ω: (**a**) conventional fixed-bandwidth ESO with $\omega_{o}=1500\;\mathrm{rad}/s$, (**b**) proposed FESO. The arrows indicate the peak undershoot and the settling time.

Figure 11a shows the output waveform when the observer bandwidth $\omega_{o}$ is set to 1500rad/s. This corresponds to 15 times the conventional control bandwidth $\omega_{c}$, which exceeds the typical range of 3 to 10 times. As the bandwidth expands, the high-frequency noise of the output voltage is amplified and reflected in the control signal, as illustrated in Figure 6a. Consequently, it can be observed that the output waveform is affected by the noise. The waveform of the proposed FESO in Figure 11b corresponds to $n_{\mathrm{VL}}=3$ and $n_{\mathrm{VH}}=15$, which define the lower and upper limits of the observer bandwidth $\omega_{o}$ as 300rad/s and 1500rad/s. The FESO operates with a low bandwidth under normal conditions to minimize noise influence and increases the bandwidth when internal or external disturbances occur, or during transient periods, according to fuzzy logic. The FESO achieves a settling time of 23ms and an undershoot of 3.2V, and compared to the ESO with a fixed bandwidth of $\omega_{o}=1500\;\mathrm{rad}/s$, the output waveform demonstrates that it is more robust to noise.

**Figure 12:**
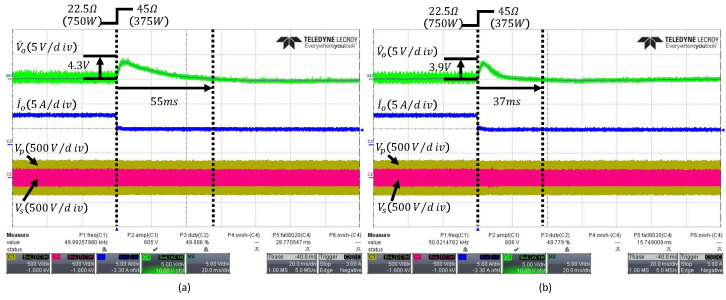
Transient responses under load change from 22.5Ω to 45Ω: (**a**) PI controller, (**b**) conventional fixed-bandwidth ESO with $\omega_{o}=300\;\mathrm{rad}/s$. The arrows indicate the peak overshoot and the settling time.

When the load changes from 22.5Ω to 45Ω, the output waveforms of the PI and ESO controller are shown in Figure 12a,b, respectively. The conventional ESO exhibits a voltage overshoot of 3.9V and a settling time of 37ms, whereas the PI controller shows a voltage overshoot of 4.3V and a settling time of 55ms, indicating that the PI controller’s transient performance is inferior to that of the ESO.

**Figure 13:**
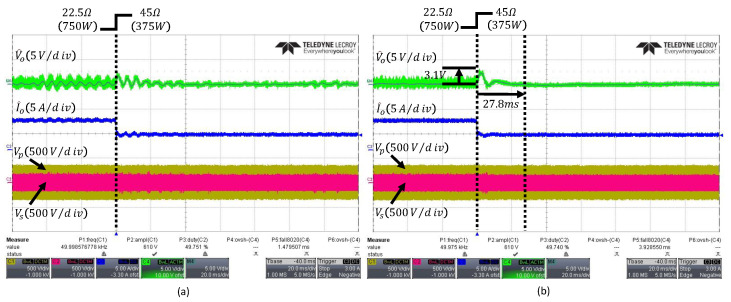
Transient responses under load change from 22.5Ω to 45Ω: (**a**) conventional fixed-bandwidth ESO with $\omega_{o}=1500\;\mathrm{rad}/s$, (**b**) proposed FESO. The arrows indicate the peak overshoot and the settling time.

Figure 13 shows the output waveform of the proposed FESO, which achieves an overshoot of 3.1V and a settling time of 27.8ms, demonstrating improved transient performance over the conventional controllers while minimizing the influence of noise.

Table 2 summarizes the output-voltage deviation and settling time of each controller under both load-decrease and load-increase conditions. From this table, it can be observed that the proposed FESO reduces both the voltage deviation and the settling time, thereby improving the transient performance.

## 6. Discussion

In this work, the proposed ESO-based disturbance compensation is implemented within the local (converter-level) digital controller of a single DAB-based ESS interface; therefore, it does not require inter-agent communication and introduces no additional communication overhead in our experimental setup. Nevertheless, if the framework is extended to networked/distributed cooperative control (e.g., higher-layer voltage restoration and power sharing), communication constraints may become an important design factor. In such cases, event-triggered strategies have been reported as an effective approach to reduce communication while maintaining closed-loop performance, e.g., the adaptive event-triggered output-feedback scheme in [31]. Exploring an event-triggered communication design for distributed extensions of the proposed local controller is an interesting direction for future work.

## 7. Conclusions

Based on the in-depth analysis of the trade-off associated with the bandwidth of the extended state observer (ESO), this paper proposes a Fuzzy-based ESO (FESO) with variable bandwidth to overcome the limitation of the conventional fixed-bandwidth approach. The proposed FESO adjusts the bandwidth using fuzzy logic, thereby minimizing the influence of noise while improving transient response performance. Experimental results show that, under load increase conditions, the proposed FESO reduces the output voltage undershoot by 22% and 20% compared to the conventional PI controller and ESO, respectively, and shortens the settling time by 48.9% and 36.1%. Under load decrease conditions, the output voltage overshoot is reduced by 27.9% and 20.5% compared to the PI controller and ESO, respectively, with the settling time reduced by 49.5% and 25%. These results demonstrate that the proposed FESO provides superior transient response performance compared to conventional controllers under severe step-load conditions, thus providing ADRC for the DAB as the bidirectional power flow for the DC microgrid.

## Figures and Tables

**Table 1:** System parameters.

Parameter	Value	Parameter	Value
$V_{i}$	300 V	$C_{f}$	1880μF
*L*	158μH	*N*	0.5
$V_{\mathrm{ref}}$	130 V	$f_{s}$	50 kHz

**Table 2:** Performance comparison for load-change tests.

	Load Increase (375 W → 750 W)		Load Decrease (750 W → 375 W)	
Controller	Output Voltage Undershoot	Settling Time	Output Voltage Overshoot	Settling Time
PI	4.1 V	45 ms	4.3 V	55 ms
ESO	4.0 V	36 ms	3.9 V	37 ms
FESO	3.2 V	23 ms	3.1 V	27.8 ms

## Data Availability

The original contributions presented in this study are included in the article. Further inquiries can be directed to the corresponding author.

## Appendix A. Derivation of the ESO Structure and Pole-Placement Gains

This appendix provides the detailed derivation of the extended state observer (ESO) used in the ADRC framework, including the error dynamics and the pole-placement gain expressions. These derivations are omitted from the main text to improve readability.

### Appendix A.1. Extended-State Representation

Starting from the ADRC lumped model,

(A1) $$y^{\left(n\right)}\left(t\right)=f\left(t\right)+bu\left(t\right)$$

The disturbance is augmented as an additional state to obtain the extended state representation:

(A2) $$\begin{array}{cc} {\dot{x}}_{1}\left(t\right) & =x_{2}\left(t\right)\\ {\dot{x}}_{2}\left(t\right) & =x_{3}\left(t\right)\\ \; & \vdots \\ {\dot{x}}_{i}\left(t\right) & =x_{i+1}\left(t\right)\\ {\dot{x}}_{n}\left(t\right) & =x_{n+1}\left(t\right)+bu\left(t\right)\\ {\dot{x}}_{n+1}\left(t\right) & =\dot{f}\left(t\right)\\ y(t) & =x_{1}\left(t\right) \end{array}.$$

### Appendix A.2. Luenberger-Observer Formulation

The ESO can be written in the Luenberger-observer form:

(A3) $$\begin{array}{cc} \dot{z} & =Az+Bu+L\left(y-Cz\right)\\ \tilde{y} & =Cz \end{array}$$

where the companion-form matrices are given by

(A4) $$A=\left[\begin{array}{ccccc} 0 & 1 & 0 & \cdots & 0\\ 0 & 0 & 1 & \cdots & 0\\ \vdots & \vdots & \ddots & \ddots & \vdots \\ 0 & 0 & \cdots & 0 & 1\\ 0 & 0 & \cdots & 0 & 0 \end{array}\right],\;B=\left[\begin{array}{c} 0\\ 0\\ \vdots \\ 0\\ b \end{array}\right],\;C=\left[\begin{array}{cccc} 1 & 0 & \cdots & 0 \end{array}\right]$$

And the observer gain is

(A5) $$L={\left[\begin{array}{cccc} \beta_{1} & \beta_{2} & \cdots & \beta_{n+1} \end{array}\right]}^{T}.$$

### Appendix A.3. Component-Wise ESO Form

Using (A3), the ESO dynamics can be expanded as

(A6) $$\begin{array}{cc} {\dot{z}}_{1}\left(t\right) & =z_{2}\left(t\right)+\beta_{1}\left(y\left(t\right)-z_{1}\left(t\right)\right)\\ {\dot{z}}_{2}\left(t\right) & =z_{3}\left(t\right)+\beta_{2}\left(y\left(t\right)-z_{1}\left(t\right)\right)\\ \; & \vdots \\ {\dot{z}}_{i}\left(t\right) & =z_{i+1}\left(t\right)+\beta_{i}\left(y\left(t\right)-z_{1}\left(t\right)\right)\\ \; & \vdots \\ {\dot{z}}_{n}\left(t\right) & =z_{n+1}\left(t\right)+\beta_{n}\left(y\left(t\right)-z_{1}\left(t\right)\right)+bu\left(t\right)\\ {\dot{z}}_{n+1}\left(t\right) & =\beta_{n+1}\left(y\left(t\right)-z_{1}\left(t\right)\right). \end{array}$$

### Appendix A.4. Estimation Error Dynamics and Characteristic Polynomial

Define the estimation error as e=x−z. Neglecting $\dot{f}\left(t\right)$ for the pole-placement analysis, the error dynamics become

(A7) $$\dot{e}\left(t\right)=\left(A-LC\right)e\left(t\right).$$

The characteristic equation is therefore

(A8) $$\det\;\left(sI-(A-LC)\right)=0$$

which yields the polynomial form

(A9) $$s^{n+1}+\beta_{1}s^{n}+\beta_{2}s^{n-1}+\cdots +\beta_{n+1}=0.$$

### Appendix A.5. Pole-Placement Gain Design

A standard ADRC/ESO pole-placement choice is to place all observer poles at $-\omega_{o}$, leading to

(A10) $$s^{n+1}+\beta_{1}s^{n}+\beta_{2}s^{n-1}+\cdots +\beta_{n+1}={\left(s+\omega_{o}\right)}^{n+1}.$$

Accordingly, the observer gains are obtained as

(A11) βi=n+1iωoi,i=1,2,…,n+1

where

(A12) n+1i=(n+1)!i!(n+1−i)!.

As $\omega_{o}$ increases, the observer bandwidth increases and the estimation error converges faster; however, excessively large $\omega_{o}$ amplifies measurement noise and may be limited by sampling and switching-frequency constraints.
